# Design Polyaniline/α-Zirconium Phosphate Composites for Achieving Self-Healing Anti-Corrosion of Carbon Steel

**DOI:** 10.3390/nano14010076

**Published:** 2023-12-27

**Authors:** Ziqi Lv, Kai Ren, Tao Liu, Yunyan Zhao, Zhonghua Zhang, Guicun Li

**Affiliations:** College of Materials Science and Engineering, Qingdao University of Science and Technology, Qingdao 266042, Chinazhangzh@qust.edu.cn (Z.Z.); guicunli@qust.edu.cn (G.L.)

**Keywords:** polyaniline, α-zirconium phosphate, phytic acid, anti-corrosion, self-healing

## Abstract

The rupture of a micro/nano container can trigger the release of repair agents and provides the coating with a self-healing and anti-corrosion effect. However, the defect and inhomogeneity of the coating, produced by the rupture of the micro/nano container, may weaken its anti-corrosion performance. This study reports a rare protection mechanism, which optimizes the space occupying of zirconium phosphate, and the de-doping peculiarity of polyaniline without the rupture of the micro/nano container. Polyaniline/α-zirconium phosphate composites were constructed through in situ oxidation polymerization. Repair agents were added in the form of doped acids. According to the different repair agents in polyaniline/α-zirconium phosphate composites (citric ion, tartaric ion and phytic ion), the performance and protection mechanism of the composites were researched. Polyaniline/α-zirconium phosphate coating (with phytic ion) shows an excellent self-healing anti-corrosive effect, due to the large spatial structure and abundant chelating groups of the precipitation inhibitor. Considering the anti-corrosive application, the developed polyaniline/α-zirconium phosphate composite has a far-reaching influence on marine development.

## 1. Introduction

Carbon steel is used extensively for marine engineering equipment [1,2,3]. Applying anti-corrosion coatings is one of the most economic and effective methods for the anti-corrosion effect in the metal in extremely aggressive environments [4,5,6]. In recent years, zirconium phosphate (ZrP), a two-dimensional layered compound, has been widely used for anti-corrosion coatings [7,8,9,10]. ZrP in coating form can distribute uniformly, fill the defects, block the corrosion medium, and further enhance the corrosion protection of the coating [11,12,13,14,15]. Nevertheless, the inevitable microcracks in service will reduce the protective effect of the coating on the metal [16]. Hence, polyaniline (PANI), one of the most promising conductive polymers, has been widely used for anti-corrosive coatings due to its passivation effect. PANI may suppress the cathode corrosion reaction by the metal surface passivation produced by its oxidizing nature [17,18,19,20]. Nevertheless, the passivation effect of PANI is not enough to greatly improve the long-term anti-corrosion quality of the coating.

Recently, anti-corrosion coatings with a self-healing function have been created to solve the problem of short service periods after coating damage [6,21,22,23,24]. Zheng et al. [25] prepared the microcapsules of multilayered shell structures loaded with dicyclopentadiene to achieve good self-healing performance. Dieleman et al. [26] used PVA electrospun fiber mats loaded with different corrosion inhibitors (CeCl_3_ and Li_2_CO_3_) and embedded in epoxy coatings for the long-term protection of relatively large damages. In the healing mechanism, the repair agent can be released from the micro/nano container when it is stimulated by the external environment [27]. Corrosion inhibitors, as highly efficient repair agents, may self-repair the damaged coatings by forming a film on the metal surface [28]. Valentini and Vaghefinazari et al. investigated the effects of different corrosion inhibitors on microstructural evolution, phase composition and corrosion behavior [29,30]. However, the healing action is limited by insufficient container space and the defect of the coating [31]. The rupture of the micro/nano container can release corrosion inhibitors, but it may also cause the defect and inhomogeneity of the coating.

Herein, we endeavor to provide a new design for container-less self-healing anti-corrosion. We make ingenious use of the doping and de-doping characteristics of PANI to hold and release corrosion inhibitors. In order to obtain excellent long-term anti-corrosion, polyaniline/α-zirconium phosphate composites are synthesized. We integrate the barrier effect of zirconium phosphate, the passivation of PANI and the container-less release of corrosion inhibitors on the anti-corrosion system. Simultaneously, a more suitable corrosion inhibitor for this system is explored. The effect of the structure of the corrosion inhibitors on the micromorphology and anti-corrosion property of the composite is also discussed. As a result, this study provides a theoretical basis for the selection of corrosion inhibitors. Furthermore, novel scheme for container-free corrosion inhibitor coatings avoids the defect and inhomogeneity of the coating, and provides more possibilities for the development of self-healing coatings. The strategy of combining the superior physicochemical properties of the fillers offers a new insight into the development of anti-corrosion coatings in marine environments.

## 2. Experimental Section

### 2.1. Materials

Aniline, hydrochloric acid (HCl) and tartaric acid (TA) were purchased from Shanghai Epi Chemical Reagent Co., Ltd. (Shanghai, China). Citric acid (CA) was purchased from Tianjin Bodi Chemical Co., Ltd. (Tianjin, China). Phytic acid (PA) was purchased from Aladdin Industrial Co., Ltd. (Shanghai, China). Phosphoric acid (concentrated phosphoric acid with a weight percentage of 85%), zirconium phosphate (ZrOCl_2_·8H_2_O), poly-methyl methacrylate (PMMA), toluene and ammonium persulfate (APS) were purchased from Shanghai McLean Biotechnology Co., Ltd. (Shanghai, China).

### 2.2. Preparation of α-ZrP

According to the literature [32], to 1.07 g ZrOCl_2_·8H_2_O was added 615 μL H_3_PO_4_ with 85% by weight (H_3_PO_4_/Zr = 3). The samples were placed in a Teflon-lined stainless steel autoclave, stirred well and heated at 200 °C for 24 h. 

### 2.3. Preparation of Polyaniline/Zirconium Phosphate Composites

The 305 μL of aniline and 0.1 g of α-ZrP were dispersed in 50 mL water using ultrasonic waves. Additionally, 4 mmol hydrochloric acid and ammonium persulfate (0.748 g) were added and the mixture was kept in an ice water bath for 12 h. To compare the effects of doped acids, hydrochloric acid was replaced by citric acid (CA), tartaric acid (TA) and phytic acid (PA), respectively. A nano-structured polyaniline/zirconium phosphate composite was obtained, which was denoted hereafter as PANI−HCl/α-ZrP, PANI−CA/α-ZrP, PANI−TA/α-ZrP, PANI−PA/α-ZrP, respectively, according to the type of doped acid. For comparison, α-ZrP was not added in the mixture to obtain PANI.

### 2.4. Preparation of Composite Coatings

**Figure 1:**
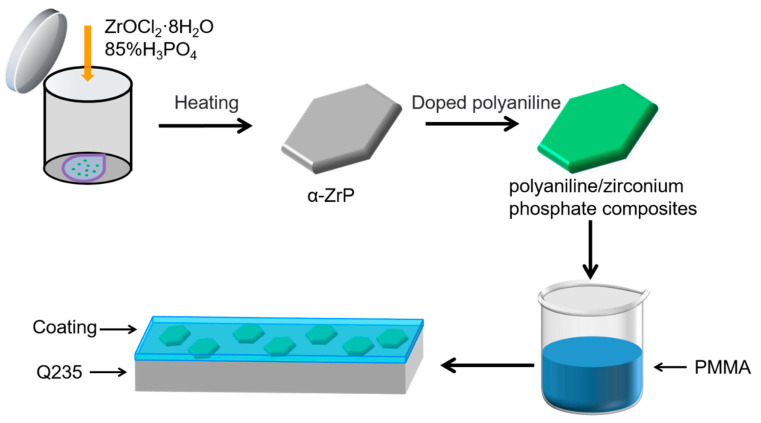
Composite coating preparation diagram.

A total of 50 mg of PANI (PANI−HCl/α-ZrP, PANI−CA/α-ZrP, PANI−TA/α-ZrP, PANI−PA/α-ZrP) was dispersed in toluene (5.25 mL) under ultrasonic agitation for 30 min. Then, 0.3 g/mL of polymethyl methacrylate (PMMA) solution in toluene was added to the above mixture, stirring constantly. After 3 h standing, P−PMMA (PHZ-PMMA, PCZ-PMMA, PTZ-PMMA, PPZ-PMMA) liquid was obtained, as shown in Figure 1. The polyaniline/α-zirconium phosphate composite coatings with a thickness of about 80 μm were prepared by drop-casting technique. The thickness of the film was controlled by the volume of the drop-casting solution. The as-prepared polyaniline/α-zirconium phosphate composite coatings were dried at room temperature for 5 days. The polished Q235 carbon steel (120 mm × 120 mm × 1 mm) coated with polyaniline/α-zirconium phosphate composite coatings were employed to carry out the corrosion studies.

### 2.5. Characterizations

The morphologies of the composites were characterized by a JSM-6700F scanning electron microscope (SEM, JEOL Ltd., Tokyo, Japan). X-ray diffraction (XRD) spectrum was recorded by a D-max-X-γA diffractometer (Rigaku Ltd., Tokyo, Japan) in the 2θ range of 5–70°. The structure and composition of composites were identified with a Nicolet iS10 Fourier transform infrared spectrometer (Thermo Fisher Scientific Ltd., Waltham, MA, USA) from 3800–400 cm^−1^. The static water contact angle was recorded using a LSA100 optical contact angle measuring device (LAUDA Scientific Ltd., Lauda-Königshofen, Germany). X-ray photoelectron spectroscopy (XPS) was recorded through an AXIS SUPRA X-ray spectrometer (SHIMADZU, Manchester, UK). The corrosion products were analyzed by an invia Qontor laser Raman spectrometer (Renishaw plc, London, UK). The coating thickness was measured with a TT260 coating thickness gauge (Time plc, Beijing, China). Tafel curves and electrochemical impedance spectroscopy (EIS) were recorded on a CHI-760E electrochemical workstation (Chenhua plc, Shanghai, China). The saturated calomel electrode (SCE), platinum sheet and uncoated and coated Q235 carbon steel electrodes were taken as reference electrode, counter electrode and working electrodes, respectively. All tests were performed in NaCl (3.5 wt%) corrosive solution. For the EIS measurements, the frequency was in the range from 100 kHz to 10 mHz with signal amplitude of 10 mV at open circuit potential (OCP). Tafel polarization tests were conducted by scanning the electrode potential automatically from −250 mV to +250 mV at a scan rate of 10 mV s^−1^. Three parallel samples were used in the corrosion test of each sample to avoid experimental errors. Salt spray tests were characterized by a YW-60A salt spray corrosion test chamber (Senyuan plc, Zibo, China).

## 3. Results and Discussions

### 3.1. Morphological and Structure Characteristics of Polyaniline/Zirconium Phosphate Composites

**Figure 2:**
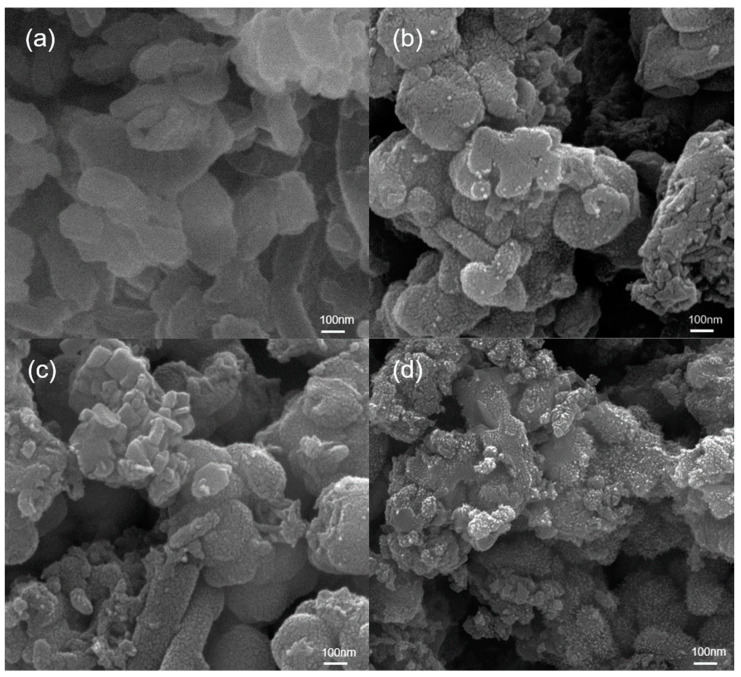
SEM images of PANI−HCl/α-ZrP (**a**), PANI−CA/α-ZrP (**b**), PANI−TA/α-ZrP (**c**), PANI−PA/α-ZrP (**d**).

The morphologies of polyaniline/zirconium phosphate composites were observed via SEM photographs. PANI−HCl/α-ZrP in Figure 2a shows the similar lamellar structure of about 200 nm with α-ZrP (Figure S1b). Precursor α-ZrP in Figure S1a has X-ray diffraction peaks at 11.6°, 19.7°, 24.9°, 33.7°, 34.0°, corresponding to the (002), (110), (112), (020), and ($\bar{3}$12) crystal plane of α-ZrP (PDF# 22-1022), respectively. The polyaniline/zirconium phosphate composites show nearly the same XRD peaks as zirconium phosphate (Figure S1a), so the composition of the composites was also characterized through FT-IR spectra. The similar morphology of PANI−HCl/α-ZrP and α-ZrP suggests the even growth of PANI on the surface of α-ZrP by electrostatic adsorption. However, the outer cladding PANI displays an increasing number of synapses, as seen in Figure 2a–d. As the spatial structure of the doped acid increases gradually, the morphology of PANI is affected, and then the morphology of the complex is affected. The different morphology of the composite is attributed to the different growth environment of micro-nano structures. Alkaline aniline is adsorbed on the surface of acidic zirconium phosphate by electrostatic adsorption, and then the doped acid reacts with aniline to form aniline salt. Salts exist as micelles in aqueous solutions. After the addition of ammonium persulfate oxidant, the polymerization of aniline will be carried out on the surface of micelles. That is to say, micelles play a template role in the formation of micro-nano structures of polyaniline. The shape of the micelle is strongly dependent on the reaction conditions. During the synthesis of the four compounds, the amount of aniline and dopant acid are the same, but only the type of dopant acid is changed. From hydrochloric acid to phytic acid, polyaniline molecules are embedded with larger-sized counter-ions. Moreover, with the increase in the hydroxyl group, the hydrogen bond interaction between hydroxyl groups becomes stronger. Moreover, the hydrogen bond interaction between hydroxyl group and amino group on the main chain of polyaniline also increases. Hence, the growth direction of polyaniline is changed and more synapses are generated. The morphology of more synapses increases the specific surface area of the complex. When the coating is destroyed and the corrosion reaction occurs, the release path of the dopant acid will be shorter. In order to investigate the self-healing effect of the inhibitor the in coating, the contents of polyaniline in different coatings were the same, and a non-inhibitor-doped acid (hydrochloric acid) was used as a control. Three common corrosion inhibitors (citric ion, tartaric ion and phytic ion) were used to compare the self-healing effects. The shorter release path of corrosion inhibitor leads to the fact that a new passivation layer can be generated on the metal surface in time. Therefore, corrosion inhibitors in the coating play a self-healing role.

**Figure 3:**
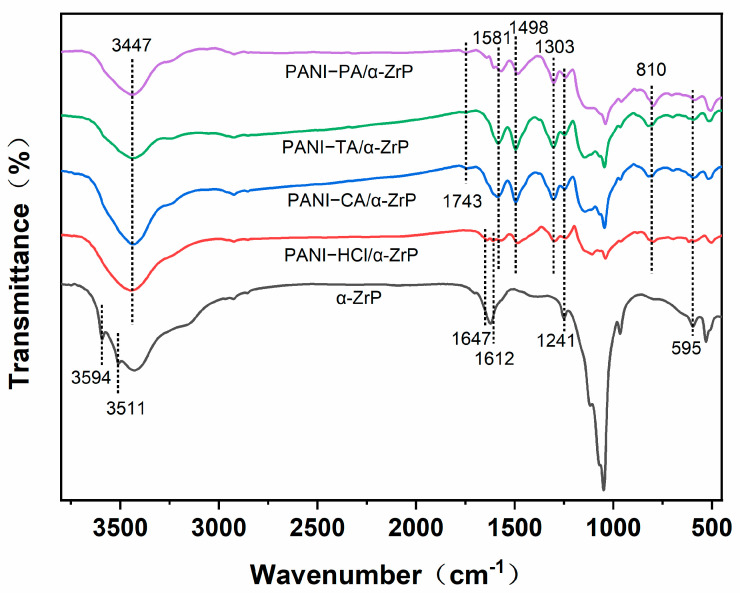
FT-IR spectra of α-ZrP, PANI−HCl/α-ZrP, PANI−CA/α-ZrP, PANI−TA/α-ZrP, PANI−PA/α-ZrP.

The FT-IR spectra in Figure 3 also prove the successful introduction of doped acid in the complex. All of the samples show P−O and Zr−O tensile vibrations (1241 cm^−1^ and 595 cm^−1^) of zirconium phosphate. Moreover, all composites display the characteristic absorption peaks of PANI. The peaks at 1498 cm^−1^, 1303 cm^−1^ and 810 cm^−1^ are attributed to the C=C, C−N and C−H stretching bonds of the benzene ring, respectively. The peaks at 3447 cm^−1^ and 1581 cm^−1^ correspond to the tensile vibration of N−H and C=N of PANI, respectively. Unlike other samples, PANI−HCl/α-ZrP reveals a stretching vibration of C−Cl (1647 cm^−1^ and 1612 cm^−1^). Other composites show stretching bonds of C=O at 1743 cm^−1^. This is due to the fact that carboxyl structures exist in citric acid, tartaric acid and phytic acid. With respect to the composite samples, the peaks at 810 cm^−1^ and 505 cm^−1^ are attributed to the C−H out-of-plane bending vibrations of 1,4-disubstituted aromatic rings. The doped imine salt structure with alternating benzene and quinone rings also indicates that various acids enter the PANI skeleton in the form of doped acids.

**Figure 4:**
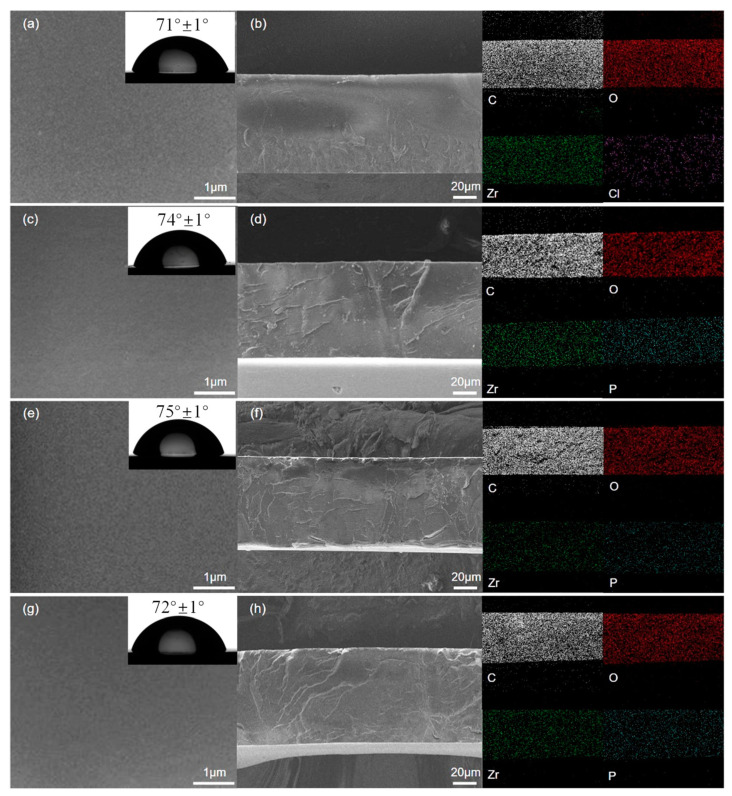
SEM images, static water contact angles (insets) and EDS mapping of different coatings. The SEM images are divided into surface views (left) and cross-sectional (right): (**a**,**b**) PANI−HCl/α-ZrP; (**c**,**d**) PANI−CA/α-ZrP; (**e**,**f**) PANI−TA/α-ZrP; (**g**,**h**) PANI−PA/α-ZrP.

Different coatings’ surfaces and cross-section SEM micrographs are presented to study the effects of corrosion inhibitors on the morphology and porosity of the coating. It can be seen from Figure 4a,c,e,g that the surfaces of all composite coatings are smooth, uniform and compact. There are no visible holes and cracks on the coating’s surface. Moreover, the cross-section micrographs of the coatings are obtained under liquid nitrogen. A fracture of the cross-section exhibits many clear folds. Similarly, the cross-section micrographs also show that the composite coatings have no holes and the coatings are dense. All coatings exhibit a similar thickness (about 80 microns). In this study, the amount of liquid coating was strictly controlled and a film thickness meter was used to further screen the coating samples. However, the static water contact angles of the coatings were different. The hydrophobic property of the PANI−PA/α-ZrP coating is slightly worse than that of the PANI−CA/α-ZrP and PANI−TA/α-ZrP coatings. This may be attributed to the many hydrophilic phosphonic acid groups of PANI−PA/α-ZrP. As shown in the EDS mapping, the composites are uniformly dispersed in the coatings. The even distribution of the chlorine element in the PANI−HCl/α-ZrP coating also proves that the dopant acid is uniformly distributed in the coating. Therefore, the change in dopant acid does not affect the porosity, thickness and density of the coating. However, the hydrophobic property of the coating is affected by the dopant acid.

### 3.2. Anti-Corrosion Performance Composite Coating

**Figure 5:**
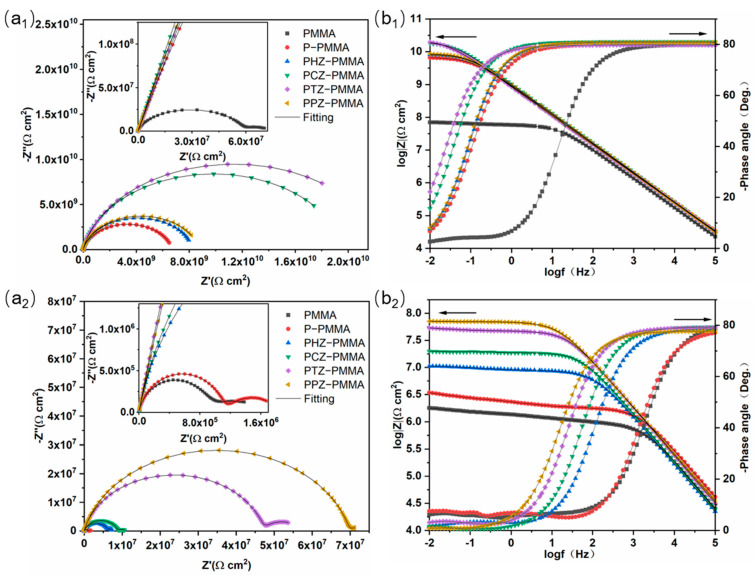
Nyquist plots (**a_1_**,**a_2_**) and Bode plots (**b_1_**,**b_2_**) of each coating soaked in 3.5 wt % NaCl solution for 24 h (**a_1_**,**b_1_**) and 168 h (**a_2_**,**b_2_**). The Bode plots are divided into Bode magnitude plots (left ordinate) and Bode phase angle plots (right ordinate). Color dots in Bode plots represent the same samples in Nyquist plots.

**Figure 6:**
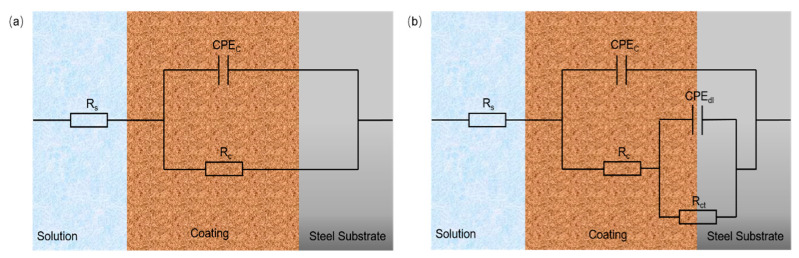
Equivalent electrical circuits (EECs) (**a**) model 1 and (**b**) model 2 used for simulation of the EIS measurements of the specimens.

In order to investigate the effect of doped acid on self-healing corrosion protection, the electrochemical impedance spectra of each coating after immersion were measured, and the fitting results are shown in Figure 5. For the sake of an in-depth exploration, Zview 3.0a software was used to fit the original EIS data (Table 1) through two equivalent circuit models, as shown in Figure 6. Among the relevant parameters of the equivalent circuit model, *R_s_*, *R_c_*, *R_ct_*, *CPE_c_* and *CPE_dl_* represent solution resistance, coating resistance, charge transfer resistance, coating capacitance and double-layer constant phase element, respectively. The inhibition efficiency (IE) of corrosion is calculated using the following formula:

IE = (*R_ct_* − *R_ct,c_*)/*R_ct_* (1)

where *R_c__t_* and *R_c__t__,c_* are the charge transfer resistance values for the resin coating with filler and control sample (PMMA resin coating), respectively. The distinction in Figure 6a,b represents if harmful chemicals reached the interface of the coating and substrate. Figure 6a represents the early corrosion stage, while Figure 6b shows what occurs in the late corrosion stage. Figure 5(a_1_,a_2_) shows the Nyquist diagram of each coating when the corrosion time is 24 and 168 h. The corrosion resistance of the coating is positively correlated with the value of the impedance arc diameter. In NaCl corrosion medium, the corrosion resistance of the coating deteriorates with the increase in time. It can be seen from Table 1 that all fillers increase the coating resistance of traditional film-forming resin PMMA at the early corrosion stage, indicating a good physical barrier property of zirconium phosphate in the coating. More intuitive information can be obtained from Bode magnitude plots and Bode phase angle plots in Figure 4b. In the early stage of corrosion, the density, uniformity and hydrophobicity of the coating are major factors affecting the impedance. As Nyquist plots and Bode plots show, the impedance of the PANI−PA/α-ZrP coating is smaller than that of the PANI−CA/α-ZrP and PANI−TA/α-ZrP coatings. The main reason for this may be the poor hydrophobicity of the PANI−PA/α-ZrP coating. As shown in Bode phase angle plots, the samples show two-time constant features (two peaks) in Figure 5(b_2_), confirming that the corrosive medium has reached the interface of the coating/substrate. At the later stage of corrosion, the factors that can influence the impedance are the passivation layer caused by the passivation effect of polyaniline and the new passivation layer caused by the release of corrosion inhibitors. It can be seen that the charge transfer resistance of the samples without polyaniline is lower than that of the samples with polyaniline due to the absence of a passivation layer. Moreover, the samples containing polyaniline showed different anti-corrosion effects due to the different doped acids. The possible chemical reactions which occur on the interface are presented as follows:

Fe → Fe^2+^ + 2e^−^ (2)

O_2_ + 4e^−^ + 2H_2_O → 4OH^−^ (3)

(PANI^y+^·yA^−^)n + nye^−^ → (PANI)n + nyA^−^ (4)

where A^−^ = Cl^−^, citrate ion, tartrate ion or phytate ions.

The doped PANI is de-doped in the local alkaline environment. For polyaniline, acid doping can be carried out under acidic conditions, and acid disappearance (de-doping) only occurs under alkaline conditions. Hence, the composites will not lose dopants, if no corrosion reaction occurs. The composites in this work take advantage of the doping and de-doping properties of polyaniline to accommodate and release corrosion inhibitor. From this moment on, impedance modulus values follow an order of PPZ−PMMA (7.11 × 10^7^ Ω cm^2^) > PTZ−PMMA (5.38 × 10^7^ Ω cm^2^) > PCZ−PMMA (1.99 × 10^7^ Ω cm^2^) > PHZ−PMMA (1.06 × 10^7^ Ω cm^2^). The PPZ-PMMA coating has the maximum IE (80.78%), which is 29.79% higher than that of PHZ−PMMA. Compared with PHZ−PMMA, other polyaniline/zirconium phosphate composite coatings display a better anti-corrosion effect. This is potentially due to the fact that the corrosion inhibitor (CA, TA or PA) is combined with the metal substrate to form a new protective layer. When the corrosion reaction occurs, the local pH value changes, and the highly efficient repair agent is detached and released from the composite coating. Subsequently, the corrosion inhibitor combines with the metal substrate to form a new passivation layer to play a self-healing role. The impedance modulus value of PPZ−PMMA is the largest among all samples, which implies that the more abundant chelate groups lead to stronger adhesion on the metal substrate. As a result, PPZ−PMMA shows the best self-healing corrosion protection.

**Figure 7:**
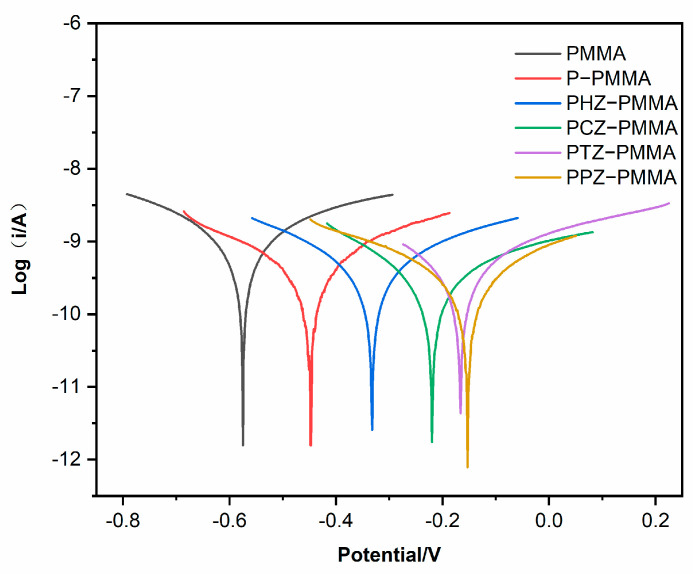
Polarization curves of PMMA, P−PMMA, PHZ−PMMA, PCZ−PMMA, PTZ−PMMA and PPZ−PMMA immersed in 3.5 wt% NaCl solution for 168 h.

The anti-corrosion properties of the coatings were further investigated by Tafel polarization tests. Tafel polarization curves, as seen in Figure 7, were recorded after 168 h of immersion in corrosion liquid. The values of corrosion potential (*E_corr_*), corrosion current density (*I_corr_*) and polarization resistance (*R_P_*) of different coatings were calculated by the Tafel extrapolation method and CHI-760E V1.0 software in electrochemical workstation (Table 2) [33]. The IE is calculated using the following formula:

IE = 1 − (*I_corr_/I_corr,c_*) (5)

where *I_corr_* and *I_corr,c_* are the *I_corr_* values for the resin coating with filler and control sample (PMMA resin coating), respectively. Compared with the PMMA sample, the positive shift of the *E_corr_* of other specimens indicates the anodic protection produced by PANI. Moreover, the *I_corr_* of PHZ-PMMA coating is less than that of the P−PMMA coating. It is proved that the addition of 2D nanomaterial zirconium phosphate can block corrosive medium from reaching the surface of metallic substrates. The corrosion inhibition efficiency calculated by the polarization curve is consistent with that calculated by impedance. Last but not least, the PPZ−PMMA coating has the maximum polarization resistance (1.826 × 10^8^ Ω/cm^2^), which is 61.28% higher than that of PHZ−PMMA. The self-healing corrosion protection of the PPZ−PMMA coating could offer distinct advantage over the traditional corrosion protection of PHZ−PMMA.

**Figure 8:**
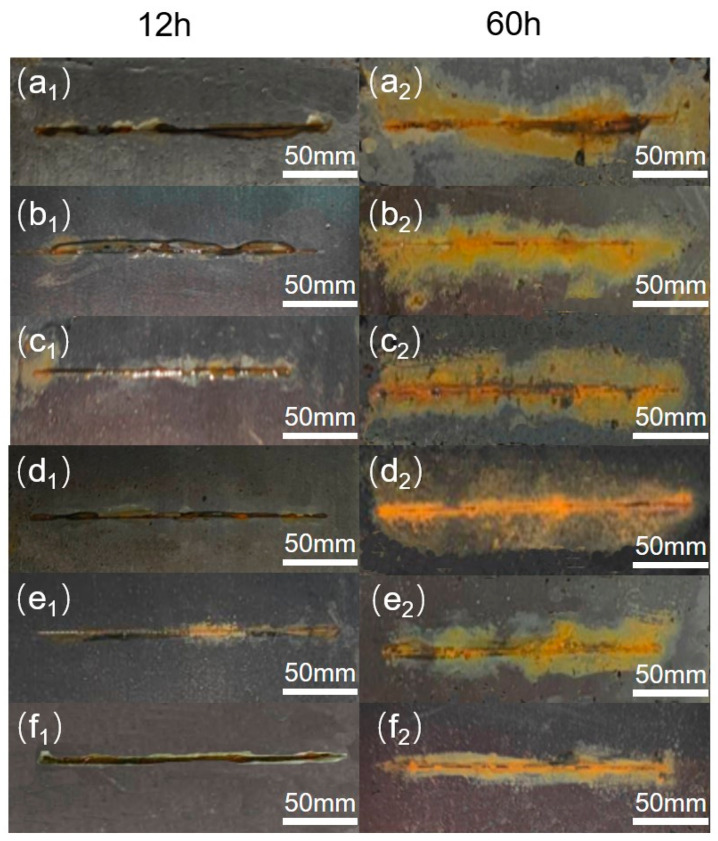
The appearance of various coatings exposed to salt spray test after 12 h and 60 h. (**a_1_**,**a_2_**) PMMA, (**b_1_**,**b_2_**) P−PMMA, (**c_1_**,**c_2_**) PHZ−PMMA, (**d_1_**,**d_2_**) PCZ−PMMA, (**e_1_**,**e_2_**) PTZ−PMMA, (**f_1_**,**f_2_**) PPZ−PMMA.

Salt spray tests are also proof of the excellent corrosion protection of PPZ−PMMA. According to GB/T 10125 neutral salt spray (NSS) test conditions, 5% NaCl solution was used for spray, and the test temperature was 35 °C [34]. Before the experiment, specimen coatings coated on metallic substrates were cut and Q235 carbon steels were exposed to air. Scratch areas were created by cutting the coating with a scalpel. The length and the width of the scratch area are about 200 mm and 0.2 mm, respectively. Figure 8 shows the photographs of specimen coatings after different hours of NSS test. From Figure 8, it can be seen that a corrosion reaction has occurred on all specimens after 12 h. However, the specimens with a corrosion inhibitor (Figure 8(d1,e1,f1)) displayed less corrosion products than other specimens. This was further evidence that the corrosion inhibitor may be effectively released and improve the corrosion protection. Moreover, it can be clearly seen that PPZ−PMMA (Figure 8(f2)) has the minimum corrosion area around the scratch zone among all specimens after 60 h. The excellent self-healing corrosion protection of PPZ−PMMA can be attributed to the abundant chelate groups of phytic acid.

### 3.3. Anti-Corrosion Mechanism

**Figure 9:**
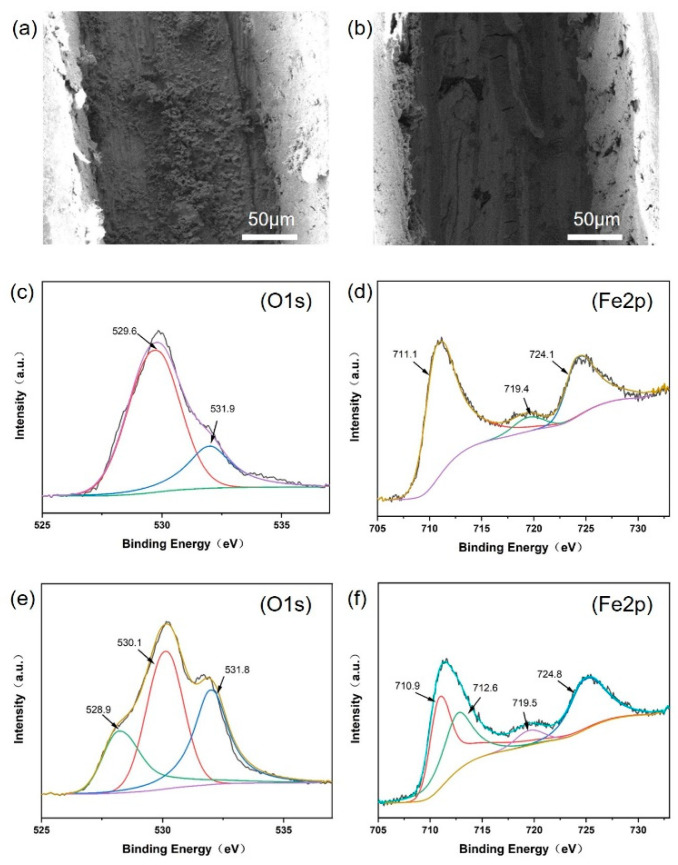
The SEM images of the scratch on the coatings immersed in 3.5 wt% NaCl solution at 24 h. (**a**) PHZ−PMMA and (**b**) PPZ−PMMA. Narrow spectra for element O (**c**) and Fe (**d**) of the scratch on the PHZ−PMMA coating in (**a**). Narrow spectra for element O (**e**) and Fe (**f**) of the scratch on the PPZ−PMMA coating in (**b**).

In order to verify the self-healing effect of the coating, the morphology and composition of the scratch zone of the coating were investigated. Figure 9a,b show the morphologies of the scratches of PHZ−PMMA and PPZ−PMMA coatings immersed in 3.5 wt% NaCl solution at 24 h. A large amount of fluffy rust is found on the surface of the PHZ−PMMA. For the PPZ−PMMA coating, a dense passivation layer appears on the scratch, which could prevent the permeation of the corrosive media. XPS measurements were performed to confirm the chemical composition of the scratch zone. Figure 7c,d show O 1s and Fe 2p XPS spectra obtained from the PHZ−PMMA sample. The O 1s spectra were fitted by two peaks (529.6 and 531.9 eV), corresponding to −OH groups of Fe_2_O_3_. The characteristic peaks at 711.1 and 724.1 eV are attributed to Fe_2_O_3_ (2p3/2 and 2p1/2, respectively), and the peak at 719.4 eV is due to the shake-up line of Fe^3+^. The conjecture from the SEM result is confirmed. For the PPZ−PMMA sample, a new characteristic peak at 528.9 eV appears in the O 1s spectrum in Figure 9e, which is ascribed to the P−O−Fe bond. Moreover, the characteristic peaks at 712.6 eV also prove the existence of the P−O−Fe bond. This result indicates the formation of a phytic acid iron passivation layer on the PPZ−PMMA sample. Therefore, the corrosion inhibitor phytic ion can repair the metal in time when it corrodes, and the production of a phytic acid iron passivation layer has a self-healing effect.

**Figure 10:**
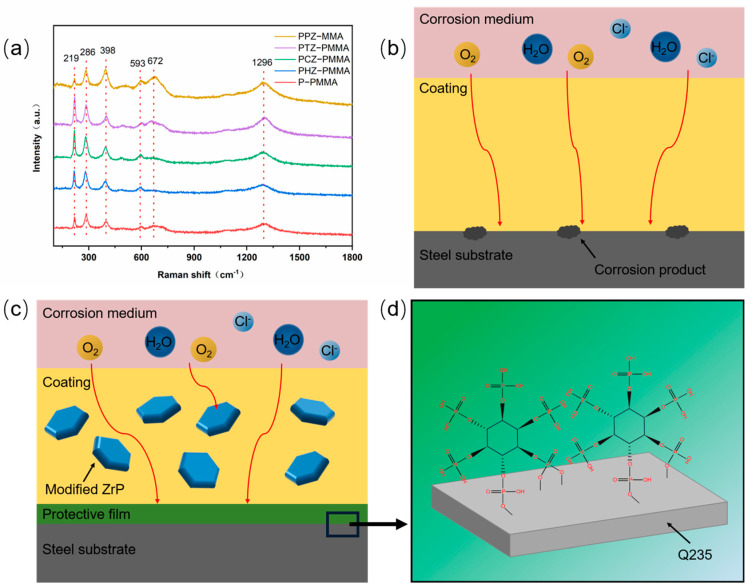
(**a**) Raman spectra of the scratched area on steel substrates after pulling coatings off. Anti-corrosion mechanism of PMMA coating (**b**) and PPZ−PMMA coating (**c**,**d**).

The main components of the scratched area were further analyzed by the Raman test after 168 h of immersion in the corrosive liquid (Figure 10a). The characteristic peak at 398 cm^−1^ is assigned to α-FeOOH, and the peaks at 219 and 286 cm^−1^ are attributed to α-Fe_2_O_3_. The rust composition suggests that corrosive medium has reached the surface of metal and a corrosion reaction has occurred. The characteristic peaks of γ-FeOOH (1296 cm^−1^) and Fe_3_O_4_ (593 and 672 cm^−1^) may be due to the passivation layer caused by the oxidation peculiarity of PANI. PANI in coatings plays two roles. On one hand, a passivation layer caused by PANI can provide a protective effect for carbon steel. On the other hand, as a switch for corrosion inhibitors, PANI composites may release the corrosion inhibitor when a corrosion reactions occur. Moreover, the existence of a precipitation inhibitor (citrate ion, tartrate ion or phytate ions) can make up for the defects of the passivation layer. The synergistic effect of dual passivation makes the passivation layer more complete and stronger.

To achieve an enhanced anti-corrosion effect, multiple protection mechanisms are designed. Considering the discussions above, we propose the possible anti-corrosive mechanism of the PPZ−PMMA coating (Figure 10c). Compared with PMMA resin coating (Figure 10b), the two-dimensional layered compound α-ZrP in the coating extends the transport path of the corrosive medium, and prevents corrosion. Moreover, the in situ growth of PANI on the surface of zirconium phosphate by electrostatic adsorption results in the increased exposed area. Therefore, the contact area with metal substrate enlarges, which is conducive to the formation of a passivation layer. In addition, a corrosion inhibitor is used to add the self-healing function. The traditional self-healing coatings release the corrosion inhibitors by the rupture of the container. However, the de-doping property of PANI is used in this paper to release the corrosion inhibitors and avoid the coating defects caused by the container rupture. The experimental results show that the elaborate design can effectively enhance the anti-corrosion quality of the coating.

Three precipitation inhibitors are compared in this research. Both citrate ion (three carboxyl groups, one hydroxyl group) and tartrate ion (two carboxyl groups, two hydroxyl groups) have four chelating groups to cooperate with metal. Thus, the better self-healing anti-corrosion of PTZ−PMMA may be attributed to the larger passivation area caused by the larger spatial structure of tartrate ion. Furthermore, phytic ion has six phosphate groups, which is a rare “metal multi-tooth chelator”. The abundant phosphate groups in its molecular structure can easily provide electron pairs to the metal with empty orbitals (Figure 10d). As a result, the phytic ion is firmly adsorbed on the surface of the metal by the formation of multiple chelating rings. The anode corrosion reaction is further inhibited as the chelation of the iron ion. Thus, both larger spatial structures and more chelating groups of precipitation inhibitors facilitate the self-healing anti-corrosion effect. The best corrosion protection of the PPZ−PMMA coating may be ascribed to multiple protection mechanisms.

## 4. Conclusions

In this study, polyaniline/zirconium phosphate composites were successfully synthesized by in situ oxidation polymerization. The designed polyaniline/zirconium phosphate wrapping structures exploit both the maze effect of the layered compound α-ZrP, as well as the passivation effect of PANI. Meanwhile, the structural design utilizes the novel release principle of corrosion inhibitors, which takes full advantage of the de-doping characteristic of PANI. As a result, the anti-corrosion efficiency of the PPZ-PMMA coating is 80.78 percent higher than that of the traditional PMMA resin coating after soaking for 168 h. Compared with other coatings, the PPZ−PMMA coating presents excellent anti-corrosion performance with a significant polarization resistance value of 1.826 × 10^8^ Ω /cm^2^. The change in dopant acid does not affect the porosity, thickness and density of the coating. However, the micromorphology of PPZ−PMMA shows more synapses due to the larger spatial structure of the dopant acid and the fact that there are more hydrogen bonds. The morphology of more synapses increases the specific surface area of the complex. When the coating is destroyed and the corrosion reaction occurs, the release path of the dopant acid will be shorter. The shorter release path of the corrosion inhibitors lead to the fact that a new passivation layer can be generated on the metal surface in time. There is reason to believe that a large spatial structure and abundant chelating groups are beneficial to the self-healing anti-corrosion effect of materials. The designed releasing mechanism of inhibitors, without breach of the micro/nano container, provides a new view of angle for the self-healing anti-corrosive coatings.

## Figures and Tables

**Table 1:** Fitting parameter of EIS tests performed on different coatings.

Time	Samples	CPE_C_		R_c_ (Ω cm^2^)	CPE_dl_		R_ct_ (Ω cm^2^)	IE(%)
		(Ω^−1^cm^−2^S^n^)	n_f_		(Ω^−1^cm^−2^S^n^)	n_f_		
24 h	PMMA	3.143 × 10^−10^	0.8879	5.8338 × 10^7^	1.403 × 10^−7^	0.6376	2.067 × 10^7^	-
	P-PMMA	2.111 × 10^−10^	0.8875	6.718 × 10^9^	-	-	-	-
	PHZ-PMMA	2.005 × 10^−10^	0.8967	8.287 × 10^9^	-	-	-	-
	PCZ-PMMA	1.830 × 10^−10^	0.9006	1.965 × 10^10^	-	-	-	-
	PTZ-PMMA	2.124 × 10^−10^	0.8889	1.975 × 10^10^	-	-	-	-
	PPZ-PMMA	1.986 × 10^−10^	0.8971	8.754 × 10^9^	-	-	-	-
168 h	PMMA	7.653 × 10^−10^	0.8105	2.031 × 10^7^	6.879 × 10^−6^	0.4376	1.119 × 10^7^	-
	P-PMMA	2.237 × 10^−10^	0.8978	2.621 × 10^7^	9.504 × 10^−7^	0.4362	2.089 × 10^7^	46.43
	PHZ-PMMA	2.312 × 10^−10^	0.9023	3.906 × 10^7^	4.135 × 10^−7^	0.4341	2.281 × 10^7^	50.99
	PCZ-PMMA	3.806 × 10^−10^	0.8784	6.406 × 10^7^	5.122 × 10^−8^	0.4821	2.531 × 10^7^	55.79
	PTZ-PMMA	2.823 × 10^−10^	0.8635	6.987 × 10^7^	2.811 × 10^−8^	0.5631	3.056 × 10^7^	63.38
	PPZ-PMMA	2.833 × 10^−10^	0.8591	7.671 × 10^7^	8.795 × 10^−8^	0.4161	5.820 × 10^7^	80.78

**Table 2:** Tafel extrapolation results obtained.

Samples	*I_corr_* (A/cm^2^)	*E_corr_* (V)	IE (%)	*R_P_* (Ω/cm^2^)
PMMA	1.298 × 10^−9^	−0.5768		3.383 × 10^7^
P-PMMA	6.863 × 10^−10^	−0.4486	47.12%	6.105 × 10^7^
PHZ-PMMA	6.106 × 10^−10^	−0.3315	52.96%	7.072 × 10^7^
PCZ-PMMA	5.595 × 10^−10^	−0.2214	56.89%	7.583 × 10^7^
PTZ-PMMA	4.676 × 10^−10^	−0.1674	63.97%	8.913 × 10^7^
PPZ-PMMA	2.416 × 10^−10^	−0.1553	81.38%	1.826 × 10^8^

## Data Availability

The data presented in this study are available on request from the corresponding author.

## Supplementary Materials

The following supporting information can be downloaded at: https://www.mdpi.com/article/10.3390/nano14010076/s1, Figure S1. X-ray diffraction of different samples (a) and scanning electron microscope images of α-zirconium phosphate (b).
