# Supplementary material for: Design Polyaniline/α-Zirconium Phosphate Composites for Achieving Self-Healing Anti-Corrosion of Carbon Steel
Source: Nanomaterials (Basel). 2023 Dec 27;14(1):76. doi: 10.3390/nano14010076 (PMC10780750; doi:10.3390/nano14010076)
Supplement: Supplementary file 1 [file nanomaterials-14-00076-s001.zip › nanomaterials-2751989-supplementary.pdf]

Supplementary Materials

Design Polyaniline/α-Zirconium Phosphate Composites for Achieving Self-Healing Anti-Corrosion of Carbon Steel

Ziqi Lv, Kai Ren, Tao Liu, Yunyan Zhao *, Zhonghua Zhang and Guicun Li

College of Materials Science and Engineering, Qingdao University of Science and Technology, Qingdao 266042, China; zhangzh@qust.edu.cn (Z.Z.); guicunli@qust.edu.cn (G.L.)
* Correspondence: zhaoyy@qust.edu.cn

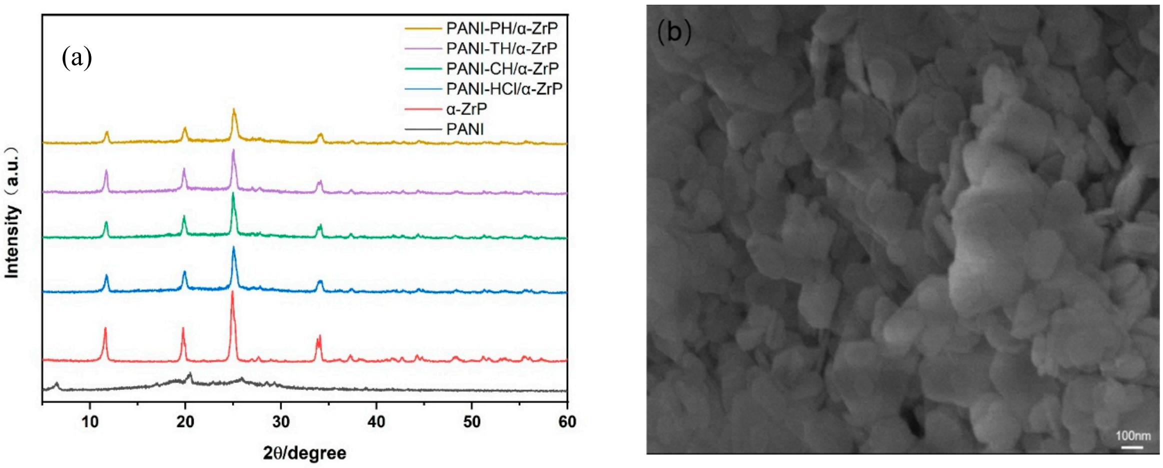

Figure S1. X-ray diffraction of different samples (a) and scanning electron microscope images of α-zirconium phosphate (b).
